# Supplementary material for: Reassessing the Abundance of miRNAs in the Human Pancreas and Rodent Cell Lines and Its Implication
Source: Noncoding RNA. 2023 Mar 17;9(2):20. doi: 10.3390/ncrna9020020 (PMC10037588; doi:10.3390/ncrna9020020)
Supplement: Supplementary file 1 [file ncrna-09-00020-s001.zip › Supplementary information-miRNA profile in pancreas-SI 031623.pdf]

Supplementary information to

1. Supplementary Tables

Table S1. Primers used in the S-PolyT method for detection of mature miRNAs. "Rev-polyT" is the universal miRNA-qPCR primer. "f-" refers to miRNA specific forward primers, "rt-" refers to reverse transcription primers. Lower case nucleotides (nts) in a miRNA-specific forward primer were added to the 5' end to increase the Tm of that primer. The last 6 nts (red) in the reverse primers were used to match a specific isoform of a miRNA. Lower case "a" and "b" at the end of reverse primers refer to the top-2 isoforms of a miRNA according to the abundance (RPM).

Name	Sequence (5' to 3')
Rev-polyT	CAGTGCAGGGTCCGAGGT
f-miR-375-3p	TTTGTTCGTTCGGCTCGCG
rt-miR-375-3p-polyT	CAGTGCAGGGTCCGAGGTCAGAGCCACCTGGGCAATTTTTT TTTTTCGCGAG
f-miR-7-5p	cccgTGGAAGACTAGTGATTTTG
rt-miR-7-5p-polyT	CAGTGCAGGGTCCGAGGTCAGAGCCACCTGGGCAATTTTTT TTTTTAACAAA
f-miR-148a-3p	cgcTCAGTGCACTACAGAACTT

rt-miR-148a-3p-polyT	CAGTGCAGGGTCCGAGGTCAGAGCCACCTGGGCAATTTTTT TTTTTAGACAA
f-miR-26a-5p	ctcgTTCAAGTAATCCAGGATAG
rt-miR-26a-5p-polyTa	CAGTGCAGGGTCCGAGGTCAGAGCCACCTGGGCAATTTTTT TTTTTAGCCTA
rt-miR-26a-5p-polyTb	CAGTGCAGGGTCCGAGGTCAGAGCCACCTGGGCAATTTTTT TTTTTCTATCC
f-miR-217-5p	gcATACTGCATCAGGAACTGA
rt-miR-217-5p-polyT	CAGTGCAGGGTCCGAGGTCAGAGCCACCTGGGCAATTTTTT TTTTTCAGTTC
f-miR-27a-3p	cgcTTCACAGTGGCTAAGTTC
rt-miR-27a-3p-polyT	CAGTGCAGGGTCCGAGGTCAGAGCCACCTGGGCAATTTTTT TTTTTGAACTT
f-miR-21-5p	cgcTAGCTTATCAGACTGATGT
rt-miR-21-5p-polyTa	CAGTGCAGGGTCCGAGGTCAGAGCCACCTGGGCAATTTTTT TTTTTAACATC
rt-miR-21-5p-polyTb	CAGTGCAGGGTCCGAGGTCAGAGCCACCTGGGCAATTTTTT TTTTTACATCA
f-miR-143-3p	cgcTGAGATGAAGCACTGTAG
rt-miR-143-3p-polyTa	CAGTGCAGGGTCCGAGGTCAGAGCCACCTGGGCAATTTTTT TTTTTGAGCTA
rt-miR-143-3p-polyTb	CAGTGCAGGGTCCGAGGTCAGAGCCACCTGGGCAATTTTTT TTTTTAGCTAC

2. Supplementary figures

Figure S1. Immunofluorescent staining of pancreatic tissue, islets, and acinar cells. In the native pancreas, islets (insulin-positive) were surrounded by acinar cells (amylase positive) (left). After digestion of pancreatic tissue, islets were separated from acinar cells by purification, as indicated by the marker insulin and amylase respectively (right).

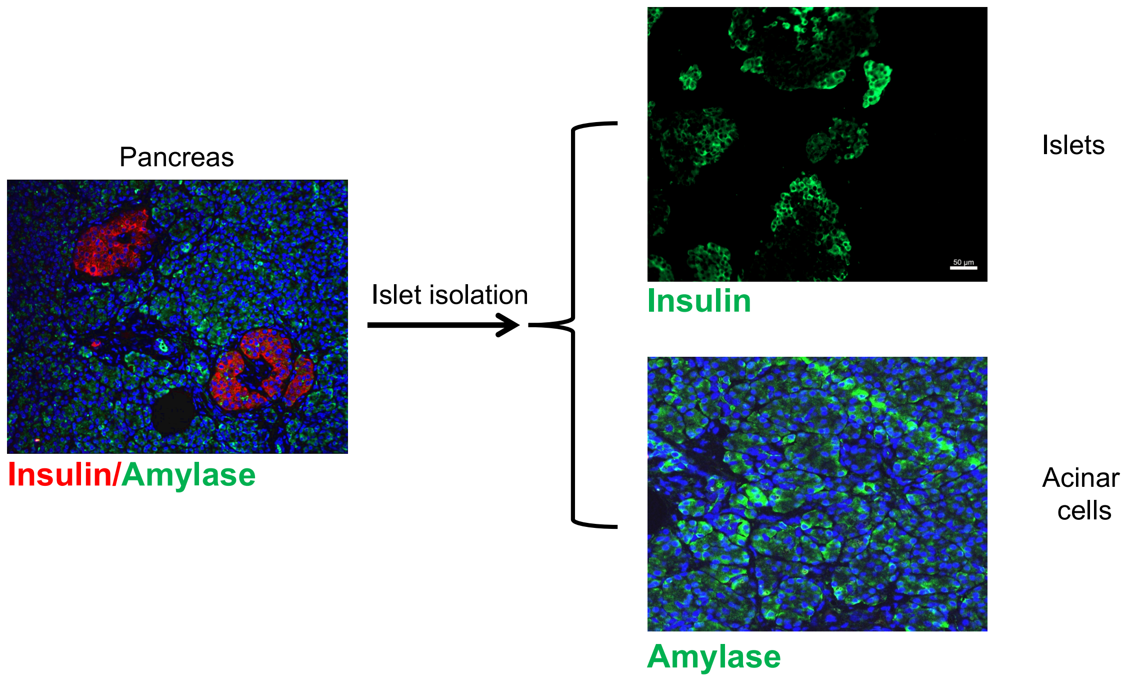

Figure S2. Characterization of the four miRge predicted novel miRNAs. (A) The four-candidate pancreas miRNAs with high read counts. (B) to (E): mFold predicted secondary structure and folding energy of the precursor miRNA (pre-miR) of the novel miRNAs.

A

Name	Tissue	Chromosome	Start Position	End Position	Strand	Mature miRNA sequence	Arm type	Read Count
miR-P1	acinus<br>islet	chr21	35720770	35720790	+	AGGAGAAUCUUUGUCACUUAG	3p	3096<br>962
miR-P2	islet	chr19	4770738	4770758	+	CAACAAGUCACAGCCGGCCUC	3p	1093
miR-P3	islet	chr2	219294165	219294185	-	GUCAUUUUUGUGAUCUGCAGCU	5p	814
miR-P4	acinus<br>islet	chrX	151959640	151959660	-	UCAGUCUCAUCUGCAAAGAAGU	3p	1088<br>833

B pre-miR-P1, bases 1 to 91, initial ΔG = -32.90

```
          10          20          30          40
GUU       U     GUC   A           A          -GUCCA
   CUGUUA UUGCA   AGU ACAAAGAUUC UCCUUGU          \
   ||:||| ||:||   ||| |||||||||| |||||||
   GAUAAU AAUGU   UCA UGUUUCUAAG AGGAACA           U
GUC       U     GAU   C           -          ACGUAC/
90         80          70          60          50
```

C pre-miR-P2, bases 1 to 90, initial ΔG = -34.60

```
          10          20          30          40
GU-     A        U  AAGA  A       U      CUGAU
   GGUCU GUGCUGUG GG    CU GUGAUUU GUUGUU     G
   :|||| |:|||:|| ||    || ||||:|| |||||:
   UCAGA CGCGAUAC CC    GA CACUGAA CAACAG     U
CCC     -        U  GGCC  -       -      CAUCA
           80          70          60          50
```

D pre-miR-P3, bases 1 to 90, initial ΔG = -48.10

```
          10          20          30          40
C     G          -            C-          --AGUAU
 UCACA CUGCCAGUG UCAUUUUUGUGAU  UGCAGCU         U
 :|||| |||||||:|| |||:||||||||:  ||||:||
 GGUGU GACGGUUAC AGUGAAAACACUG  ACGUUGA         C
C     G          U            AU          CCUCACU
           80          70          60          50
```

E pre-miR-P4, bases 1 to 91, initial ΔG = -41.60

```
          10          20          30          40
AUU  U          AA  GU        G  A        UUUGU
   GC AAGCACUUAC  CU  UUGCAGA GA ACUGAGAC     \
   || ||||||||||  ||  ||||||| || ||||||||
   CG UUCGUGAAUG  GA  AACGUCU CU UGACUCUG      A
AAC  U          AA  --        A  C        UAUCA
90         80          70          60          50
```

Figure S3. miRNA profiling in rodents. (A) Reanalysis of published miRNA profiling in murine samples. Pie chart plot of miRNAs with read counts over 1% of total miRNA read counts. (B) Reanalysis of published miRNA profiling in murine islets. Pie chart plot of miRNAs with read counts over 1% of total miRNA read counts. (C) Bar plot of highly expression miRNAs in murine alpha-TC1, beta- TC-6, and MIN6 cells. (D) Pie chart plot of reanalyzed published highly expressed miRNAs in murine MIN6 cells. (E) Bar plot of highly expressed miRNAs in rat INS1 cells.

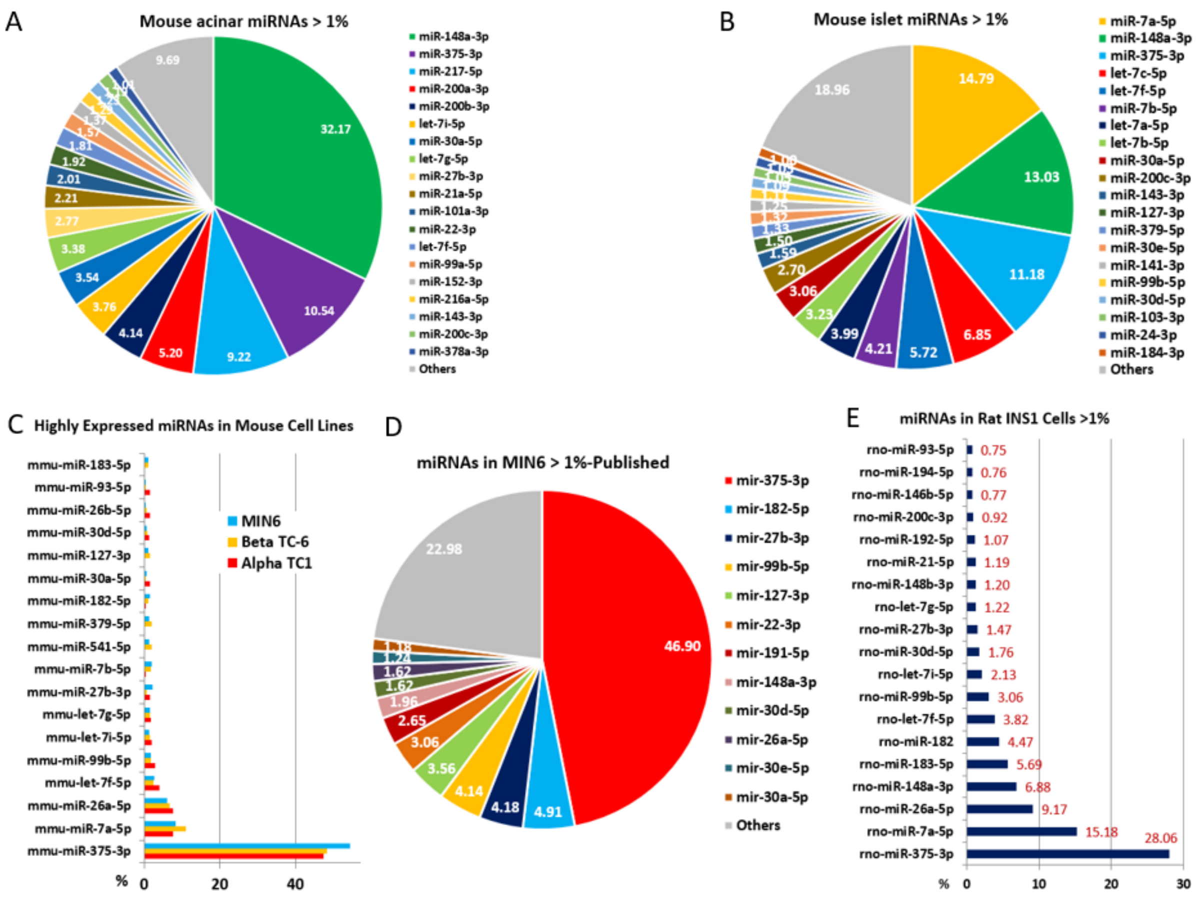

Figure S4. KEGG pathway view of islet-genes are experimentally validated targets of miR-148a-3p, miR-375, and miR-7-p. All islet-genes that are experimentally validated targets of (A) miR-7-5p, (B) miR-148a-3p, or (C) miR-375 were input into Pathview to generate KEGG pathway graph of insulin signaling pathway (hsa04910). If the node of a protein is labeled in white, no targets from the three miRNAs is involved in regulating this protein. Nodes labeled from yellow to green to red color indicate that increased number of targets are correlated with the protein at this node in the pathway.

A.

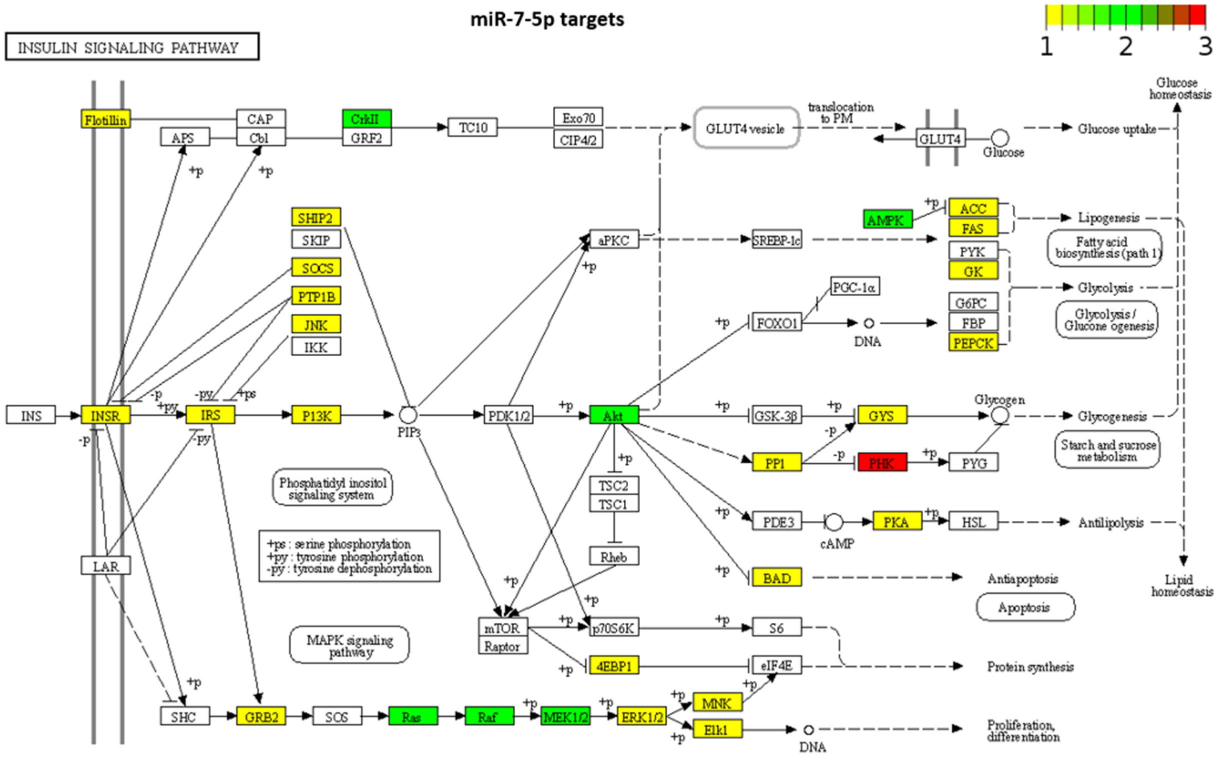

B.

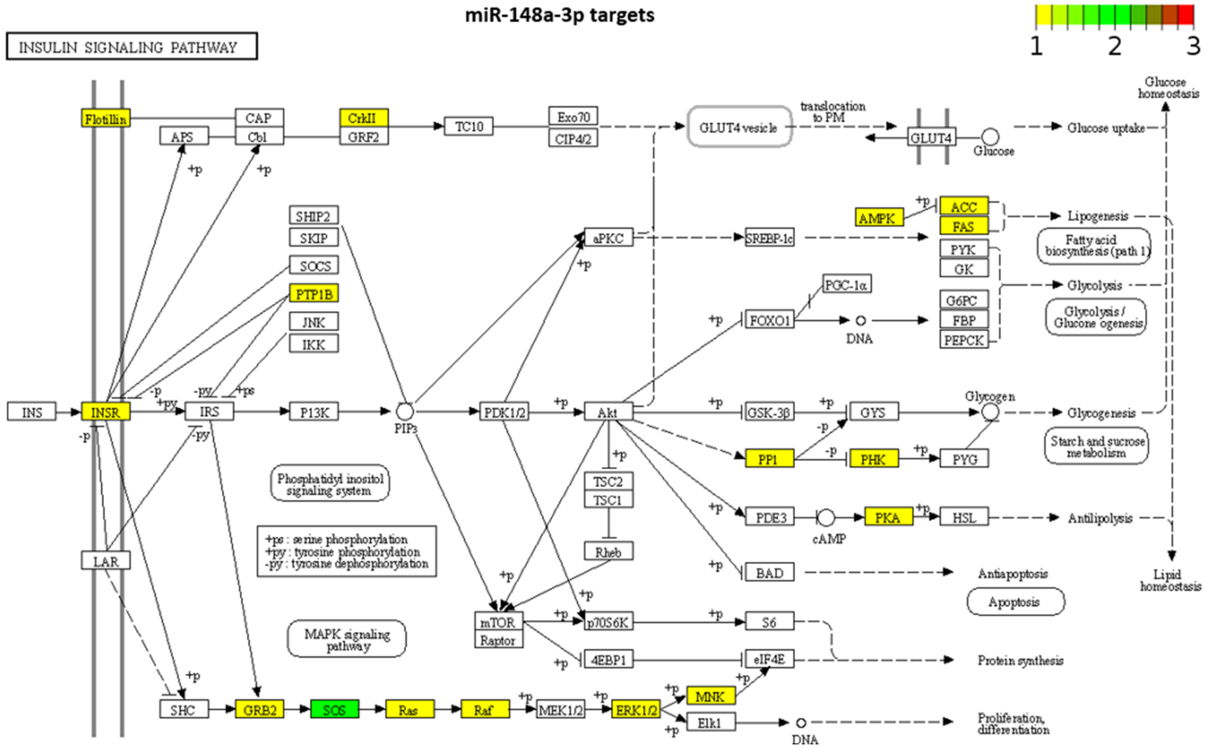
miR-148a-3p targets
1
2
3
INSULIN SIGNALING PATHWAY
Flotillin
CAP
Cbl
APS
CrkII
GRF2
TC10
Exo70
CIP4/2
GLUT4 vesicle
translocation to PM
GLUT4
Glucose
Glucose uptake
Glucose homeostasis
SHIP2
SKIP
SOCS
PTP1B
JNK
IKK
aPKC
SREBP-1c
AMPK
ACC
FAS
PYK
GK
Lipogenesis
Fatty acid biosynthesis (path 1)
PGC-1α
FOXO1
DNA
G6PC
FBP
PEPCK
Glycolysis
Glycolysis / Gluconeogenesis
INS
INSR
IRS
PI3K
PIP3
PDK1/2
Akt
GSK-3β
GYS
Glycogen
Glycogenesis
PP1
PHK
PYG
Starch and sucrose metabolism
TSC2
TSC1
Rheb
PDE3
cAMP
PKA
HSL
Antilipolysis
Lipid homeostasis
Phosphatidyl inositol signaling system
+ps : serine phosphorylation
+py : tyrosine phosphorylation
-py : tyrosine dephosphorylation
LAR
BAD
Antiapoptosis
Apoptosis
MAPK signaling pathway
mTOR
Raptor
p70S6K
S6
4EBP1
eIF4E
Protein synthesis
SHC
GRB2
SOS
Ras
Raf
MEK1/2
ERK1/2
MNK
Elk1
Proliferation, differentiation

C.

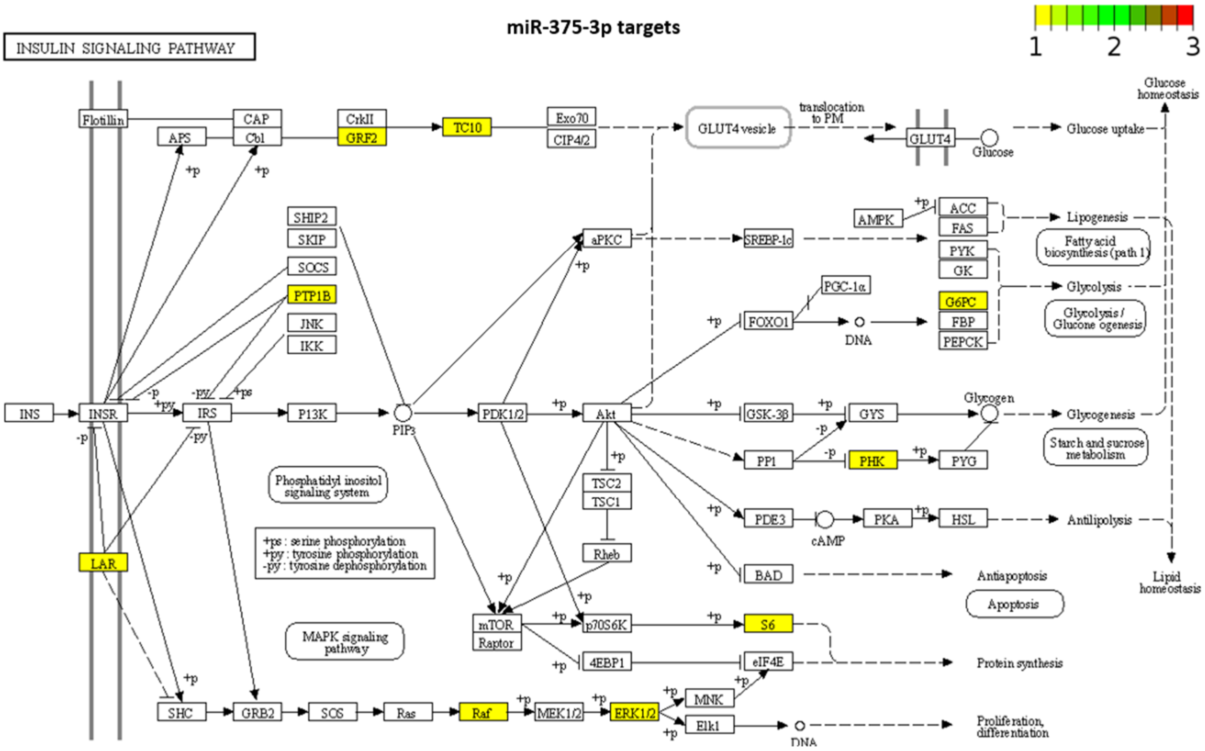
miR-375-3p targets
1
2
3
INSULIN SIGNALING PATHWAY
Flotillin
CAP
Cbl
APS
CrkII
GRF2
TC10
Exo70
CIP4/2
GLUT4 vesicle
translocation to PM
GLUT4
Glucose
Glucose uptake
Glucose homeostasis
SHIP2
SKIP
SOCS
PTP1B
JNK
IKK
aPKC
SREBP-1c
AMPK
ACC
FAS
PYK
GK
Lipogenesis
Fatty acid biosynthesis (path 1)
PGC-1α
FOXO1
DNA
G6PC
FBP
PEPCK
Glycolysis
Glycolysis / Gluconeogenesis
INS
INSR
IRS
PI3K
PIP3
PDK1/2
Akt
GSK-3β
GYS
Glycogen
Glycogenesis
PP1
PHK
PYG
Starch and sucrose metabolism
TSC2
TSC1
Rheb
PDE3
cAMP
PKA
HSL
Antilipolysis
Lipid homeostasis
Phosphatidyl inositol signaling system
+ps : serine phosphorylation
+py : tyrosine phosphorylation
-py : tyrosine dephosphorylation
LAR
BAD
Antiapoptosis
Apoptosis
MAPK signaling pathway
mTOR
Raptor
p70S6K
S6
4EBP1
eIF4E
Protein synthesis
SHC
GRB2
SOS
Ras
Raf
MEK1/2
ERK1/2
MNK
Elk1
Proliferation, differentiation

3. Supplementary data files

File_S1_List of essential pancreatic genes.xlsx: the list of essential pancreatic genes used to analyze miRNA targets

File_S2_Human islets and acinar cells miRNA count.xlsx: miRge analyzed raw read counts of miRNAs in individual human islet and acinar cells samples: "i" indicate islet samples and "a" indicate acinar cell samples.

File_S3_Novel miRNAs in human islets and acinar cells.xlsx: all miRge predicted novel miRNAs in human islets and acinar tissues

File_S4_DEseq2_results_condition_islet_vs_acinar.xlsx: DEseq2 analyzed differential expressed miRNAs in islets versus acinar cells

File_S5_Rodent cell line miRNA read count.xlsx: miRge analyzed raw read counts of miRNAs in rodent cell lines

File_S6_Pancreas cell genes that are TargetScan predicted targets.xlsx: TargetScan predicted targets that are genes expressed in pancreas

File_S7_ Islet-genes and essential genes are validated targets of miR-375, miR-7-5p, and miR-148a-3p
