# Supplementary material for: Reassessing the Abundance of miRNAs in the Human Pancreas and Rodent Cell Lines and Its Implication
Source: Noncoding RNA. 2023 Mar 17;9(2):20. doi: 10.3390/ncrna9020020 (PMC10037588; doi:10.3390/ncrna9020020)
Supplement: Supplementary file 1 [file ncrna-09-00020-s001.zip › supplementary files_03162023.zip!/supplementary files_03162023/S7-Islet-genes and essential genes are validated targets of miR-375, miR-7-5p, and miR-148a-3p.pdf]

Essential genes are validated targets of miR-375, miR-7-5p, and miR-148a-3p

Names	total	elements
miR-148a-3p miR-375 miR-7-5p	2	IRS1 SOX4
miR-375 miR-7-5p	1	GATA6
miR-148a-3p miR-375	1	PSIP1
miR-148a-3p miR-7-5p	1	ERRFI1
miR-375	9	LOXL4 PLCE1 ERBB4 HMGB3 RFX7 EGR3 RFX3 NKX6-1 EHF
miR-7-5p	10	RGS4 EGFR IRS2 CPA4 IDE IGFBP4 KRT18 PIR KRT7 PAX6
miR-148a-3p	9	PTGER3 BHLHE41 LEPR MEIS1 RGS2 PGR LCORL MAFB HHEX

Names	total	elements
Essential genes miR-148a-3p miR-375 miR-7-5p	2	IRS1 SOX4
Essential genes miR-375 miR-7-5p	1	GATA6
Essential genes miR-148a-3p miR-375	1	PSIP1
Essential genes miR-148a-3p miR-7-5p	1	ERRFI1
Essential genes miR-375	9	PLCE1 ERBB4 RFX3 NKX6-1 EHF LOXL4 HMGB3 RFX7 EGR3
Essential genes miR-7-5p	10	IRS2 CPA4 IDE IGFBP4 RGS4 PIR KRT18 PAX6 EGFR KRT7
Essential genes miR-148a-3p	9	PTGER3 LEPR RGS2 LCORL MEIS1 HHEX BHLHE41 PGR MAFB
Essential genes	99	SYT13 NEUROG3 HEX FOXA2 HNF6 NEUROD1 PDLIM4 KRT19 TFCP2L1 PROX1 CPA1 DLK1 LMO3 CRYBA2 BCHE MEIS2 SOX9 NPTX2 PPY GC GSH1 HNF1A NKX2-2 GLIS3 INS GCK RFX5 MIST1 LDB2 HNF1B CPA3 TGFBR3 PAX2 GHRL ETV1 PCSK1 REG3A LMX1A SMAD9 SIX2 PAX4 RFX1 ARX IRX2 PRG4 TTR GABRG2 GCG PCSK2 AMY2A BMP5 KLH41 SHH RBP4 SLITRK6 ADCYAP1 PFKFB2 POU3F1 NKX6-2 PTF1A CARTPT FRZB HNF4A IGF2 UCN3 FEV IAPP MAFA HES1 NOVA1 ID4 NKX2-3

		ZNF503 ISL1 HES3 ID2 GSH2 LMX1B GATA4 MNX1 THSD7A GLUT1 ENTPD2 SIX3 SOX17 PTGFR SMARCA1 CHN2 SERTM1 RFX2 IGFBP2 TM4SF4 RFX6 NKX6-3 AQP3 AIRE CDKN1C PDX1 SST

Islet-genes and pancreas essential genes are validated targets of miR-375, miR-7-5p, and miR-148a-3p

Names	total	elements
Essential genes miR-148a-3p miR-375 miR-7-5p	1	SOX4
miR-148a-3p miR-375 miR-7-5p	110	CCNI CAND1 CEP350 SON TRIP12 HSPA8 CNOT6 CBX6 RBBP6 IDS TTC3 GRPEL2 C1GALT1 ZKSCAN1 VEGFA MYC PANK3 TNRC6B NUCKS1 RCOR1 YWHAG KMT2D HECTD1 TNRC6A BIRC6 MDM2 KLHL15 DAZAP2 NDRG1 TNPO1 RAB6A CFL2 PRPF8 SKI DNAJC8 MED13 THBS1 STAU1 DDX21 CNIH4 SHOC2 LRRC8B SMG7 CDKN1A PREPL IGFBP3 SFT2D2 AKAP11 KDSR SRRM2 KMT2C INSIG1 CYP1B1 NUFIP2 AGFG1 SETD2 MRPS35 DICER1 PAFAH1B1 FEM1B UBLCP1 KIF5B NUP153 CSDE1 JAG1 UHMK1 USP9X MTF1 NRBP1 DYNLL2 AFF4 RAF1 ARPP19 CACUL1 STAT3 SPEN CRIM1 PRRC2C DCAF7 TFRC TXNIP RSBN1L YWHAB PARP1 SLC7A2 SEC23A TBC1D4 PTPN1 SESN3 HERPUD1 CUL5 SP1 H6PD SRSF1 PPIF IGF1R IPO7 UBAP2L CHD4 CAB39 RIT1 HSPH1 MAP1B MLEC CCND2 USP22 CBX5 LGALS8 WNK1 WASF2
Essential genes miR-375 miR-7-5p	1	GATA6
Essential genes miR-148a-3p miR-375	1	PSIP1
Essential genes miR-148a-3p miR-7-5p	1	ERRFI1
miR-375 miR-7-5p	185	EDN1 SH3BP4 SOD2 ERV3-1 OSBPL8 NAT10 LMNB2 MTRR CTDSP2 RBPJ SRSF6 CCDC43 UBR3 DESI1 TOR3A HNRNPR PTP4A2 NFIL3 GMFB AHCYL1 AHR NMNAT2 HNRNPUL2 CHAMP1 SERP1 SVIP EIF2S1 CRCP XIAP VEZT ARPC1A ATP6V1E1 PLOD2 RDH11 KIF1B MBD2 SNX29 NME4 PEX11B TRIP11 CHTOP HMGCR PNISR CHGB VPS35 EIF2AK2 KDM5B IP6K2 SEC16A SLC35F5 KRT80 CTNNA1 CBX4 TMED8 NIF3L1 ABL1 CMTM4 NCL SYT11 RAB32 RAB22A NUMB SSR1 PKIA TAF3 SP3 ZNF106 GLS RPL35A SLC7A1 C5orf22 SKIL HMGCS1 RPN2 ANXA7 NARS2 SEC24A LPCAT1 NUDT21 ACSL4 RCN2 VGLL4 LTA4H EIF4EBP2 SMARCD1 ILF2 SFPQ TGM2 DIP2C PHF3 ZBTB38 MAPKAP1 REEP3 MYO5B MYH9 SECISBP2 TAF2 ANKRD28 USP10 MED8 CLDN2 IFFO2 PRMT2 ZNF609 PRDX1

		ARID5B TCF12 CNN3 PPTC7 LITAF EMP1 PROSER1 SERINC2 PITPNA MTHFD2 SUB1 EFCAB14 YY1AP1 VLDLR CGGBP1 VIM CLDND1 TBC1D20 MAT2B S100A6 NFIC WDFY1 MCL1 PRRT3 IL6 EIF4G1 CASP3 XPR1 AGO1 FOSL2 INSIG2 OSTC ERP29 DDIT4 PGRMC1 BMPR2 SERPINE2 HOMER2 PCGF5 ADAMTS1 NQO1 SEC24C MXI1 SPTLC2 MAP3K2 RAB3B HNRNPDL CALU ENSA RPA1 ICAM1 NOB1 RASD1 NIPAL3 STK35 PA2G4 CALM3 SRSF2 MEST JAK1 SETD7 ADIPOR2 ZFHX3 YAP1 ELL2 ZNFX1 DCTN5 PHF10 GATAD2B TFPI2 TBC1D9 TAOK1 TMF1 PRKCA TMEM106B SKA2 DPYSL3 ATP2B1 BIRC3 DNAJC10
miR-148a-3p miR-375	83	RAB3IP ARF6 CHD1 SLK USP7 NIPBL SPTBN1 LDHB ARL2BP AP3B1 SET FAM193A AP2B1 ZBTB41 MAPK1IP1L SMAP1 PHAX KPNA4 SFXN1 MTMR4 BAZ2B PHACTR2 KLF5 SUZ12 CLCN3 MTPN SLC25A36 CBX3 PBXIP1 NONO NCOA2 RASSF8 RAB10 TPR PHLDB2 ERC1 RNPEPL1 NPDC1 UGCG TOMM20 SYNE2 TBC1D5 ATP1B1 WDR1 SLC2A3 MRPL28 RAB14 CHD6 COIL HSPA4 ENAH FBXW2 CLDN7 ZC3HAV1 STK38L MRPS7 ZBTB7A TMED5 TNS1 PPP1R10 CLTC HSP90AA1 STX3 BCL2L11 BPTF SENP2 TNPO3 REEP5 DDX6 SGMS2 HIF1A DR1 WTAP MLLT10 FYTTD1 GGNBP2 PAICS TOR1AIP2 SETX LARP4B WAC MAL2 RNF6
Essential genes miR-375	6	PLCE1 NKX6-1 LOXL4 EHF HMGB3 RFX7
miR-148a-3p miR-7-5p	322	CMTR2 BTG2 AHDC1 ESYT1 DCAF5 EP300 TRIB3 MED13L TEAD1 ARL6IP1 TSC22D1 CDC25B BAZ1B PPP1R15B KPNA2 PDIA3 DCP1A PRDM2 DNAJC5 MSH6 FRS2 GRB2 TPM3 ATXN7L3B TUBB OTUD4 KIAA0100 SRSF11 DUSP5 UBXN4 CELF1 SEC23IP CNOT1 CCT8 KDM2A TMEM14C ZBTB18 TSN SPRY2 NFYA PBX1 PPP1R12A ANKRD52 C6orf62 CCT4 ZNF146 VMP1 MRTO4 TMEM248 NSD1 LDLR DDI2 DNAJB4 EPS15 FDFT1 B2M UBE2D3 TAOK2 TEX2 CNOT7 FOXN2 KDM6B STRN3 NR1D2 RABL6 UBE2K TUFM GLCE WDR82 FAM104A IGFBP5 LIX1L GHDC ZDHHC17 PLBD2 ABHD5 MKNK2 TFAM PUM2 VKORC1L1 MAPK1 GOLGB1 C5orf51 PTBP3 CAPNS1 ICMT EFHD2 EEF1D PACS1 FZD5 TRAK2 NEO1 VPS37A TMEM181 BLCAP EML4 PPP2R2A CPEB4 TMBIM6 C16orf72 PPP1CB TSPYL1 SNRK ATP2B4 RB1 MT-ND5 ERLIN1 MAP3K4 PSKH1 TMED10 PPRC1 ADIPOR1 PDE4DIP NACC1 UBE2Z COL4A1 TM4SF1 GINM1 PURB KBTBD2 LURAP1L RAB11FIP5 FBXL3 CNOT8 HELZ PTPRJ RPL11 VMA21 ARHGAP35 DUS1L GNB1 ADNP CALD1 EPAS1 UBAP2 PLA2G12A APC HMGXB3 GALNT1 RBM25 B4GALT5 FBXO28 MAGED1 CALM1 UBR4 ARL8B NKIRAS2 C1orf43 GOLPH3L ZNF652 LBR LPP DHCR24 PLAU NID1 MATR3 RASSF3 SEC23B GPR107 RAB8B FASN FARSB BASP1 VPS26A ACACA SQSTM1 HPS6 SCOC LNPEP

		NAPG CDK12 APOL6 RBBP9 KLF16 FYCO1 RBM23 CDKN1B ACBD5 CKB PHC3 ARF4 CLPTM1 MEF2A COPZ1 SLC25A44 BNIP2 KRAS DSG2 PPIG TUSC2 PRKAA1 RCC2 POGK FAM171A1 KLF6 ASPH YY1 FLOT2 CDK6 EIF2S3 ZNF281 TMEM14A SF3A2 COPS2 PTK7 PRKAR1A SCD DENND4B SLC38A2 WDR36 FAM168B ADAM10 TMEM214 TGFBR1 ZNF217 KANSL1 LRPPRC SETD5 PNRC1 LENG8 POLR3C ARFIP2 ALDOA MRPS30 MAZ ZNF629 PTAR1 SNAPIN ATF7IP KDM3B CHAC1 ZCCHC14 FXR1 NDUFC2 NCKAP1 WDR47 TPD52 RTN4 FBXO21 GRB10 SLC11A2 DEDD TBC1D13 MGAT2 MLLT6 GPR27 PPM1B EIF5 SLC7A5 DIP2B MBD6 HCCS CLINT1 ZKSCAN8 PSAT1 FOS TP53INP1 TUBB6 BAZ2A SECISBP2L CCNG1 MED1 HNRNPM AP3M1 ATP8A1 JARID2 USP34 APLP2 TMEM164 TMCC1 TM2D2 SRPRB TGOLN2 PIGS HSP90B1 MOB1A TRIM44 KMT2A POLR2H GBF1 TMEM242 CRTC3 INSR TGFBR2 AKAP13 BCCIP DYRK1A SLC38A1 MCM3AP RAB11A PKM CANX HIPK2 NPTN COL6A3 FOXP1 CTPS2 SUDS3 FZD6 SYVN1 DUSP16 HDGF TRMT10C STX16 UBE3C PEG10 ETNK1 KPNA6 PNN C3orf38 CRKL SESN2 SCAF11 TADA2B FLNA BAX FDPS TRIB2 HIPK1 MAT2A MDM4 PATL1
Essential genes miR-7-5p	9	PIR RGS4 IRS2 CPA4 PAX6 KRT18 KRT7 IDE IGFBP4
Essential genes miR-148a-3p	4	MEIS1 MAFB BHLHE41 RGS2
miR-375	381	CNBP COX7A2L LMAN1 CHD8 GDF15 TPRG1L DDX5 FNBP1L ARRDC2 CEACAM6 TOB2 MAP3K3 TNS3 PPCS IPO9 C4orf3 LHFPL2 WDR89 EIF4G3 ACSL3 FAM98A RTF1 EIF1 B3GNT7 GCC2 SPPL3 CDH10 SLC6A6 EIF3M EBPL SNX25 UNC13B ZBED1 PHLDA1 SEC11C NCAM1 RPL32 DIDO1 AGTPBP1 MTR PRPF3 PSMF1 YWHAZ OPA1 MOB1B SH3D19 TMEM25 NUAK1 GPBP1L1 KCTD10 C1QBP TIMM17A HNRNPA0 BEX2 MTMR12 ITGB1 RPN1 KRT8 SPAG9 CREB3 CORO1C LRP5 MAP1A NELFCD CTNNB1 FIS1 GAS8 CD200 PRKDC NACA KIAA0232 RRAGC BCAS2 CEBPZ PODXL LRIG1 MAP2K4 APEX1 ENOPH1 ZBED6 WDR26 SLC9A3R2 PTPN9 ZFP1 NNMT NCOA6 PLXND1 ARMCX3 UCHL3 YBX1 INPP5F FAM3C TMEM178B RB1CC1 EXT1 FOXJ3 CRABP2 EMC2 GTF2H1 INSM1 RPS6 KIAA0513 RTN2 HNRNPA2B1 GADD45A DMXL2 DHX8 MMP14 EIF3A SPOP PTPRF CYB5B SESN1 APPL2 MSRB2 TMX3 WDR74 PCNP PURA SAMD11 DHX38 CNTN1 BOD1L1 SLC25A27 TIMM23 SYNCRIP RBBP8 CAMSAP2 CMAS NCOR1 MTDH HSP90AB1 ARF5 PMPCB TGS1 PHLDA2 RHOQ REPS1 NSFL1C XRN1 POLR2A OGFOD1 LUC7L3 HAT1 THRA LRRC1 P4HA1 DDX1 HERC5 GDE1 SH3BGRL3 GTF2I PTPRM APH1B ZMIZ2 SLC46A3 HOOK1 AZI2 RNMT CLDN1 MRPL42 TPRKB RAB28 UBE3A DEK

		MKRN1 NCOA7 LIN7C DOCK7 FAM20C BCL10 CHPF PEBP1 AOX1 EEA1 KTN1 PROCR USP8 SYT4 RRAGB STARD7 ZBTB40 YPEL2 RHBDD1 IFIT3 RLF ACTR3 GPHN ATRX MYO1B THEM6 CCDC59 CCDC12 ECHDC1 NDST1 IFT46 DNAJB1 SERINC3 CHSY1 FBXO8 PTPN12 STX2 SLC44A2 CYP2U1 ABHD4 WDR43 USP24 USP16 ADAMTS9 AGPAT3 PTGES3 ITSN2 BBS10 AEBP2 RHOA EMC3 DCTN3 SMC1A C11orf58 EGR1 TRIM37 CDR2 ZFP36L2 GOLGA1 PCLO RRP36 AP2A1 CTSS SNAP25 MORF4L2 RAPGEF1 DLG4 SOX12 MED11 WWC1 FKBP10 SMAD7 GATAD1 BZW1 GDAP1 PEX13 COX20 ZCCHC7 MYH10 PCYOX1 AP5M1 CPM SIPA1L3 NDUFB5 H19 DRAM1 CDC42 PTER ZNF644 FLNB CRLF1 TSNAX SIN3A GBA TRIB1 PELI1 MT1X UBE2B JAZF1 MKRN2 PSME4 RAN LIMK2 PNLIPRP2 FAM200B KIAA0040 GFM2 PPP1R2 MSL2 SREK1IP1 PRDX3 LMAN2 HMGN5 ODF2 RBM12 TMEM45B EPB41 TMEM150C PRPF4 DOCK9 BRAP EBAG9 MOSPD3 NSF PLK2 NUS1 DNAJA2 PSAP SMURF1 ZFP91 INHBA IFI6 LGMN SNPH JMJD6 ELAVL4 CYP20A1 GTPBP4 LEPROTL1 RAB3D DCUN1D4 SDC1 IQGAP1 IFIT1 LIMCH1 PAFAH2 FIP1L1 ZNF841 SLC4A1AP AKAP9 DNAJC2 EFNB2 PCM1 ABHD3 MAPK3 KIAA1191 TMEM131 SPC25 STAP2 TRRAP DUT ANPEP ERP44 MGAT4A PHF14 SSTR1 TMX1 BAZ1A TSPAN1 ACIN1 YLPM1 SLC30A5 DEPTOR NUP54 ZMYND11 CDC123 NT5C2 CUL2 ARHGDIA CDV3 ZFR NOLC1 PPP2R3C FSTL3 UGP2 PABPC1 MALAT1 SLFN5 SUPT6H DERL1 F3 FOXO3 KLF10 ETV6 GEM YTHDC1 JUN PARD3 TGFBI ANP32B ZNF33A PHTF2 GOLGA4 G6PC3 NCOA4 FKBP1A TMED4 CCDC115 TTC1 SRF USP1 C11orf54 TTC7B PUM1 TRAPPC6B EXOC6
miR-7-5p	1287	FSTL1 ACTN4 PNMA1 CIPC TFIP11 PINK1 ERAL1 VPS4A LRRC41 RNF38 KLHL2 U2SURP GLTP RPS18 TMSB10 G3BP1 GALNT3 PAK1 UBAC1 MRPL10 C1RL DAZAP1 GYS1 PAIP1 C15orf48 TAF9B C2orf49 IMP4 TRIM33 NDUFS1 TMX4 CHD9 PAPOLA MSH3 RNF14 GDA AGPAT1 FXYD6 INIP SRPK1 PI4KB RRP7A MTMR1 OAZ2 SORL1 HMGN1 SLC25A14 EPS8 TANGO2 HOOK3 TRIQK ROCK2 MT-ATP6 IMPDH2 TERF2IP FOXK2 SMC3 OGDH RBM17 ALDOC ACBD3 SEL1L3 SUPT4H1 RHOC ALDH3A2 BCLAF1 EXOC3 RAD23B ABR MGAT4B RING1 NPC2 SSU72 DMTN ALDH18A1 RASGRF1 RNF4 POLE3 F2RL1 CETN3 EP400 PTK2 PPM1K OTUD3 CRELD2 YME1L1 COPS4 GCLM EIF1AX PLCL2 WDFY2 ID1 PIK3CB RAB3A ERLIN2 PCK2 FUS CXorf56 TSPYL4 ASS1 WDR19 TMEM200A POLG CEP120 POGZ HMGN4 CDC42EP3 RANBP6 PBDC1 LRRC8A RAB5B PIP4K2B CAPN1 DDHD2 PRRC2B RGL1 ZNF687 CCDC181 KIAA0355 ZNF395 KDM6A OFD1 PDP1 GLIS2 POLDIP3 ATF7 VPS25 VTCN1 GCA SLC35A5 GLA ZNF655 MRPL15 ARAP1 PICALM MARK3 DHX15 AGPS UBE2L6 STK40 IPO13 ITPRIPL2 TMED9 PJA2 ANKLE2 OSBP ALDH1L2 CEP85 RPRD1B PHF1 C11orf68 FUBP1

		AHNAK PERP FN1 RSU1 LACTB2 ASH1L BCAP29 ACO2 COQ10B IGSF8 ZNF275 FAM83H UNC5CL JADE1 HDAC3 RPL10 KRCC1 POLD2 PPM1H LSM3 LAMB3 PLSCR4 ASXL2 NCOA3 SPCS3 CUL4B YWHAH TOR1AIP1 FAU RDX BCKDK CTSB ZFP36 PXN SIRT2 DDX39A GANAB DNAJC11 SMG8 C1orf198 R3HDM2 TCP1 BRCC3 TRAM2 ERCC1 YPEL5 LXN GTF3C5 CREBBP SCRN1 RBM26 PARK7 TUBA1A EIF3D COL5A2 SNX13 SNRPC NTN4 FAM120A SH2B1 LMAN2L RFNG RAB5A MED25 SPP1 HBP1 PHGDH ICA1L BDP1 GNS MKLN1 ENO2 CBX1 TALDO1 NF1 ARFGEF2 REXO1 GALNT2 CBS TMEM69 ANKRD17 ATXN7 TRIM56 UBE2J1 HNRNPK ELF4 ANO10 ITGA6 OXA1L CCND1 NOTCH2 IMPA2 SMCHD1 PSMD12 NET1 BMP2 UBE2Q2 SURF4 GLOD4 GIGYF2 GCH1 TMOD2 FAM107B TMEM106C PIM3 SRRT RABGGTB FAM160A2 ANKRD40 CDK16 BSDC1 SAYSD1 PHF12 STX5 SPRYD7 HIPK3 EPB41L3 XPOT WSB1 ZNF480 DHCR7 SOCS4 PSMB8 PPP6R2 PPM1E CDS2 VAT1 MPDU1 ASB1 RPS4Y1 LCMT2 TOMM22 PLS3 RUVBL2 ARID1B VCP LZTS3 CALM2 SH3GL1 AUH PIP5K1C SLC25A39 ZBTB4 GNA13 GPD1L ABHD14B TSPAN31 TNFAIP3 C6orf120 ARL8A NAA40 MRPL37 KIF5C CCDC28A TMEM205 WWP1 SFXN3 SAMD5 PPP4R1 MAN1A2 SEC61A1 SERPINA4 UPF1 CRTAP IRF2BPL LSAMP TRIM47 UBE2QL1 PES1 FGFBP1 WFS1 COMMD8 ZNF165 DUSP23 CD14 TRIP6 SNAP29 IFT74 HLCS EI24 AAAS ABCD3 GSR INPPL1 ATP6V1F PTTG1IP CNN2 VAC14 COL4A2 SRCAP ARHGAP32 BMF GLO1 MRPS10 FNDC4 TRPM7 PKIG SIAH2 ATP11B RPLP0 TMBIM1 RMND5A MMP3 TRAF6 AAR2 MYL12B CSNK1E PIGH XPO6 C6orf89 KDM7A C9orf72 ZDHHC7 NAMPT ENTPD6 HACL1 SPPL2A FAM102A TXLNG PNMA2 AGRN RNF167 PRMT5 DXO ATP9A G3BP2 CLIC4 PSMB7 PPP3R1 GNAZ SCG2 TRIM4 RBM14 FASTKD5 SPTY2D1 YRDC SEC24B CXCL2 RAB2B VPS4B ZDHHC3 SLC25A4 ARHGAP1 MED7 ZFP36L1 CAPN2 TWISTNB SNX9 NECAP1 TTC39A ST6GALNAC4 RPL15 TRAPPC11 DCLRE1A RPS3A DDX3Y SRD5A1 GLT8D1 STAG1 NAP1L5 ABHD13 ZHX2 APOOL ZBTB21 ZDHHC20 PFDN1 CCNB1IP1 TMEM43 AMMECR1L PSMD6 TUSC3 SLC35A2 CCNT1 CD74 TCERG1 EHD4 DENND6A BCAT2 RTCA CBX7 RAB4A ESCO1 FARSA ACSL1 MAP2K7 UBE2H SLC6A8 TMEM38B CWC22 C15orf39 TAF11 SLC3A2 RSBN1 EIF4EBP1 PSMD10 PPP1R9B GTF2B GSPT1 SSR2 EXTL3 TMEM97 CDK5RAP3 YTHDF2 GGCX SPTBN2 BHLHE40 RNF111 IST1 NPLOC4 TRIM28 PJA1 LYSMD3 RNF24 SLC29A1 RHOG MRPL27 FBXW7 CDK2 IRF2 OSBPL9 CDC37 PPP2CA RAB11FIP1 YIF1A GRINA CLIP4 TFPI TMEM87A DDX17 FILIP1L EMC6 ESRP1 PEG3 DNTTIP2 TIPRL AZIN1 RSPRY1 ISOC1 UBE2A CDC42EP4 TM9SF3 TMEM123 RNF139 ALDH1A3 MAFG BICD2 C14orf119 STK11 CHP1 VASP RELB SDE2 KIF3A PTGFRN ZMYM2 GUCD1

		EHD1 EID2 MARCKSL1 SCAF1 LAMP2 RPS6KA1 TMEM59L LAMC2 EIF2B1 PAK4 RNF10 FAM168A ADAM17 MRPS21 IMPA1 UBE2D4 FAT1 ZC3H4 ARPC5L TCEAL3 NUDCD3 RPL37A PPP1R11 NDRG4 N4BP1 RPP25 BNIP3L SV2A FAM210B STXBP1 CUTC LSM2 ADAT1 GTF2E2 TTC14 KLHL5 CD47 SRM CRYL1 RPP25L SOCS5 IFIH1 ASXL1 CHURC1 XRCC5 KLC2 SNX12 SH3RF1 ZHX1 SIRT1 SWI5 ABCC1 HAX1 NUP205 NEK9 RNF181 AFTPH BTD AIG1 LARP4 NSMAF DDX39B TMEM192 RNF114 SFMBT1 ZNF207 TXN2 TAF15 RNF11 EFR3A ZNF703 MLH1 DEXI PHB IFNAR2 CYB5A ZNF91 PITPNC1 LPIN1 RSF1 TMEM184C GAPDH MLLT11 SBNO1 GNA12 CDC42EP1 POLR2E ATP6V0E2 TRAFD1 TNPO2 CTTNBP2NL MTCH2 ME1 ROR1 LBH ACTG1 NFU1 SLC25A23 USP39 HNRNPU SCAP RHOBTB1 EPM2AIP1 RPS9 MAP7D1 DDX56 COL1A2 MAFF HNRNPA1 AKT3 GIGYF1 KAT7 RELL2 PHKB RANBP9 SLC17A5 XPO7 REPIN1 CAPZB SAV1 LETMD1 SLC19A2 CPSF2 HDLBP FNBP1 CKAP4 RAP1GDS1 PLOD1 BAIAP2L1 GPAA1 HMCES ERGIC1 VAMP2 MEF2D VAMP8 NBN IER2 MTA2 RAP1A ID3 SLC25A37 RECQL SMARCC2 NDUFA6 SMARCC1 GNAI3 TRMT61B DSP DPYSL2 UNC5B SDHB C5orf24 RNF41 EIF3B AK3 MCRS1 UBE2E3 MED29 DDOST SLC39A14 DOLK CRY2 GPR137B MECP2 ACTL6A LYRM1 MAP3K5 SLC23A2 SNIP1 TIMP3 STAG2 CRLS1 PAIP2 TMEM127 ARHGEF12 CD55 UAP1L1 CSGALNACT2 DACH1 PTPN11 SMAD3 KDM5C ATF3 RSL24D1 ZBTB22 ANKRD10 COQ7 ZDHHC16 MDH1 DPH3 DPP4 SKP1 THYN1 PGRMC2 UBE2I GGA3 LPGAT1 BRMS1 MFAP3 SLC25A1 ARL5B SRI SOCS6 HLA-E MYO1C YWHAE SGPL1 KIFAP3 SWAP70 C5orf15 SYT5 GSTA4 ARNT MRGBP PI4KA LSM14A EIF2B3 DDAH1 PQBP1 EDC3 TIPARP SPRYD4 DTX3L FNIP1 LRRC58 NABP2 ATP13A3 COPG1 FOPNL DCAF12 CLK2 TTYH3 IDH2 SH3GLB1 SPOPL CDK2AP2 KCNH2 COMMD7 CAMK2D TEX261 DYNC1LI2 ZMYND8 KIF16B PRKD3 HSPB11 NRIP1 SPTAN1 ARL4C RCAN2 EMG1 NCEH1 FBRSL1 PPP2R1B DHX40 NUPR1 SLC1A5 DSC2 LSM10 TP53INP2 SMYD5 BCL9L TAPBP ATP2C1 AHCY NRAS MAPK9 EPB41L1 DYM ABHD15 NLGN2 RPS19BP1 PBRM1 FBXO33 SSR3 HECTD4 MAGT1 RNF20 ZBTB43 FTL DYRK2 MEA1 THBD PIK3C2A NEK7 DNALI1 IFITM2 HUWE1 PFN2 FAM222B ARPC5 ANAPC7 UBR5 STUB1 DDX42 SERPINB2 PITHD1 PRR14L LRIG3 VAPB CSK VBP1 STOM PLS1 CNRIP1 ZYX AKR7A2 LARP1 MAP2K2 SDC4 MPI MEPCE LGALS3BP S100A14 MRFAP1L1 ANGEL1 AP1M1 LDHA RPL22 EGLN1 FAM167A MRPL20 RAB6B NR1H2 PHC2 GPN3 CAPG TRIM26 CCDC86 ATN1 ORAI1 TMUB2 MPP2 TNK2 ZSWIM8 IFNGR2 NDUFA4 ASCC2 SLC44A1 SLPI CNNM4 MYO5A ADO GALC CHST14 PAK3 RSAD1 RGCC HK1 MIDN SZRD1 FAM53C BAD TPM1 SNX2 ATF5 PYCR1 MFSD10 CRTC2 MEGF9 PAPPA2 EDF1

		CYB5D2 HNRNPUL1 OSTM1 MCAM AQR NDFIP2 ADAMTSL2 DDA1 GALNT7 ISG20L2 CISD2 KIAA2026 MAN2A1 STRAP GOLGA5 UBXN2B VDAC1 TJP1 CSE1L PER2 ARFGAP2 TMED2 LYPLA2 U2AF1 SLC35B3 G0S2 FCHSD2 RRP1B DKC1 CXXC5 NELFB GID8 PPP1R12C ATG9A TCEAL4 GREM1 QPCT BCOR PFDN5 CMTM6 GOPC ILF3 PNPLA8 SLC35C2 CCNT2 KEAP1 GOSR1 PICK1 DCDC2 JOSD1 SEMA4B WASF3 TADA1 LEMD3 TXLNA UBAP1 SLC35E2B SOAT1 TMEM184B MIEF1 HEG1 SIGMAR1 CCL2 KPNA1 NUDT3 TMEM41B LAMC1 M6PR S100A11 CLASP2 SRP9 HEBP2 RPRD2 PBX3 ENKD1 GRAMD1A RAB17 PTBP1 KIF3B FAM131A PSME1 ETF1 TOPORS UBE4A ZC3H18 LYPD1 SF1 CREBL2 IDH3A LSM7 SEL1L RNF213 KCTD20 GOLGA7 DNAJC6 C2CD5 SLC35A4 LGALSL PSMA4 ZC3H13 ARPC2 PHACTR4 FOXP4 TAX1BP1 GAPVD1 NAPA NUDT2 IVNS1ABP RGS5 UHRF1BP1L GIT1 OGT P4HA2 NIPSNAP3A LRIF1 NBEAL2 HSPA13 TNC SCYL2 IFITM3 DPP3 ZNF580 WDR11 TUBB2A TTLL12 LRP6 SERPINH1 TMEM65 TLE1 DVL3 PLXNA2 SERTAD3 ANKEF1 CD59 CAPZA1 ARHGAP18 PTPRN2 EIF1B ZNF512 LSM6 BACE1 STX17 ACADSB POMP ELK1 DMKN MAPK6 DNASE2 UBE2R2 JKAMP FPGS ORC2 NUBP1 FAM136A TRIM21 AKAP1 FOXRED2 YBEY TPP1 POLR2K ZBTB33 CHST9 RPS15A ZDHHC9 PPME1 MTURN ANKRD13C MBNL1 RUSC1 FADD ABI1 DDIT3 IFIT5 SLC1A4 ENY2 ANKFY1 WIPI1 ENTPD4 RWDD1 XPO1 LATS1 RALA GNAQ GNPNAT1 PDZD8 CXCL12 DPH2 GLUL TRAF4 C19orf12 COMMD10 SACM1L CD63 EPHX1 IAH1 KLHL8 SLC20A1 SLC27A4 UBA52 FAR1 HSPBP1 SF3B1 BCL2L1 HECTD3 EIF2A SCARB2 NAV1 CHIC2 PLAGL2 ADK SLC9A1 PLEC SMG5 CA2 DNAJB9 ASNS WBP2 MLST8 CDKN2AIP CXCL1 NOL6 GPN1 SLC30A6 TCIRG1 RABEP1 SH2B3 CTDSP1 RELA DBN1 AP3D1 TSEN34 LRRC59 SLC41A2 KHSRP EDEM1 TGFA FBL IBTK TRIP4 SNX3 EIF3L FLRT2 BAP1 DZIP3 FLAD1 RAB5C PIP4K2C MLF2 AKT1S1 TSC22D4 ERF ARL3 SURF6 AAGAB EIF2AK4 NEDD8 CTDSPL MRPS2 MAP9 THUMPD1 DPP8 C6orf136 ACTB RPL23 ANKRD12 SDHC ATF6 UBTD2 EIF2AK1 TXNDC11 RANBP10 C11orf24 ACVR1B STAT2 UBA5 ALG3 RNF40 LLPH ORAI2 TBC1D10B NFX1 ZNF337 TMEM219 OXSR1 TNFAIP2 AMOTL1 MTSS1 KIAA2013 ANGPTL2 RANGRF SNX1 CRK TCF7L2 BRPF3 MORN4 DDX23 LY6E GPD2 AUP1 CETN2 IARS2 NRCAM ZMIZ1 DESI2 CHERP AKT1 ANKIB1 TMEM258 SCAMP2 TSR1 HSD17B12 GMNN FAM114A1 SERINC1 RNF149 ARF3 RAB27A PEX19 PON2 UNC119 PSMD7 SSX2IP CDC42SE2 RAD21 CKAP5 ENC1 MAP2K1 ARL15 CLIP1 CNPPD1 GTF3C6 LPCAT3 SF3B4 GTF2IRD1 MIF4GD MORN2 P4HB RBM27 COL6A1 COX4I1 OAZ1 MFSD5 BLOC1S6 SGCE IL11 F11R STRN4 HSD11B1 GSK3A GIPC1 RNF19A YKT6 KCTD12 COL5A1

		PPP2R5E GPT2 ADPRHL2 PMEPA1 DCTD EIF4G2 PLEKHM2 FICD VTI1B NUDT15 KPNB1 CCDC92 ARAF PSME3 PSEN1 BCAT1 HGSNAT MFHAS1 CSRNP2 DPAGT1 PPA1 SSBP1 HMGA1 ERN1 ATIC TMEM101 C1orf116 DDX41 UBE2D2 SNAI2 ATP1B3 TRIM25 POLR3D SCG5 NUDCD1 RNF128 CUTA PTPN13 SNAP91 SLC52A2 RPL37 DAPK1 IDH3B TDP2 ANO6 EIF3G FOSL1 ALDH9A1 SLC18B1 MISP FMN2 TRIM8 BTG3 PAK2 ANXA11 TOB1 TCEAL1 PRKAG1 LANCL1 UBE2J2 ATRN SGPP1 NRSN2 SHISA5
miR-148a-3p	478	KCNMA1 SMARCD3 ITGA5 TKT ABCB7 HSPB1 DECR1 SLC25A3 ITPK1 RAB1B DDB1 CYCS TLN1 MMP7 USP47 MIER1 KCNK3 MRPL45 MET PIKFYVE PTCD3 MAPKAPK2 ATXN10 ACTR8 ITGA3 PAPSS2 PHF20 CLTA GNAS TSPAN13 FITM2 ARHGEF9 MFF STAU2 NAT8L RIPK4 ZDHHC6 YTHDF1 TIAL1 MAOA EMP2 NRARP HSPD1 N4BP2L2 COQ9 NOP9 YWHAQ CBLL1 PPM1A ALCAM ZNF24 GNA11 BRK1 IRF3 MAPRE1 LTBP1 NDUFA2 APPBP2 KDM5A EZR DYNC1H1 ABHD12 TMEM33 GNG12 LEMD2 LYSMD2 IMMT QRICH1 MED14 PCCB PNPLA6 SPG11 TIAM1 FHL1 PPP1R37 RBM38 ATP1A1 SH3PXD2A NEK6 USP4 CCDC6 BRD4 MTMR6 PAFAH1B3 FBXO46 CSRP1 TNKS2 PGAM1 TMED7 TLK1 AGO2 DSTYK PSMC4 SGK1 GPATCH8 TSHZ1 TM9SF4 NSMF DCAF11 ISCU MCFD2 IL13RA1 RPS29 PTGS2 CCDC90B MAP4K3 NAV2 ATP6V1A C11orf49 SC5D PSMD2 IPO5 ANP32A MGP ACLY CDK4 PRMT1 CHMP3 DMXL1 METTL16 PDE3A UQCRQ SREBF2 ENPP2 MAST4 KCTD2 SAR1A DAG1 BROX VPS37B ATG3 CA12 STAT1 TWSG1 SLC2A1 SSR4 NAP1L1 USP38 QSOX2 PPARD HERC1 PTPN23 ZNF664 WDTC1 OBSL1 TTC19 SLC12A7 GFPT1 SMAD2 MAF1 UBN1 TBCEL API5 ANKH POLR1B GTF2A2 GNAI1 MRPL13 CMC2 POLDIP2 NDUFV1 BTBD1 TGFBRAP1 STAT5B TBL1XR1 ACVR1 TTI1 TXNRD1 STIP1 CCT3 HSPE1 ATXN1L ARHGAP5 MPV17 KTI12 CCDC85C MSMO1 SNF8 RC3H2 RAP2A LENG1 PPP6R3 MNT ALKBH5 ELOVL1 SUCO TAB3 ZNF512B CUL7 GORASP2 CTNND1 ELAVL1 EIF4H PPM1G ELMSAN1 TUBB4B SMARCAD1 FNDC3B NFKB1 CD164 TMEM167B CTSA UBFD1 RPL23A ELP2 PTPRU COLGALT1 MPRIP TRAPPC3 CPT1A CERS6 HLA-C SLC39A1 PSME2 ADNP2 ARCN1 TSPAN17 INO80 CNOT11 TBC1D23 FAM13B MAP2K3 SESTD1 CSNK2A1 SIK1 CCT6A SF3B3 NDEL1 REEP2 ARID1A SHMT2 NCLN KAT2B LYPLAL1 ABLIM1 TMEM54 ACKR3 PVR HMGB1 SENP5 DARS2 NAT14 KHNYN PANX1 WASL SOS1 KMT2E PTEN ATP6V0D1 AFF1 ARVCF UGGT1 TIA1 TOPBP1 AP1G1 HMG20B AMBRA1 MSN EPDR1 NRP1 GNAI2 FUBP3 GLUD2 SLC35F6 RALY CMIP HMOX2 ATP8B2 CNOT4 PCYT1A ATP11A OSMR GNPDA1 BTRC COPA PSMB6 TTLL1 ANAPC13 PPP6R1 ZNHIT1 SOS2 GPI RAI14 SGCB GDI1 TSC22D3 ARF1 BCL2L2 ATG4B PAN2 VPS41 MYO10

		SUN1 OLFM1 CASD1 TTBK2 RNF103 TMEM165 RPS8 PLAGL1 USPL1 VPS51 KDM1A FURIN SLC16A9 RAB23 CALR SNRPD1 NAA50 ITGAV ABCE1 REV3L PDPR HLA-A PPP2R5C CTBP1 TMEM9B ANKRD11 CAP1 DUSP1 RAB11FIP3 LIPA NAP1L4 NOC2L ZNF134 ZSWIM6 PPIB ATG13 TBP VAV2 GRAMD4 TES ELMOD2 AKAP17A TRAPPC10 ADRM1 RAB31 DCAF16 GTF3C2 PRRC1 DIS3 ATL3 HNRNPH3 SPIN1 PRNP ZMAT3 SNX7 MCCC2 HEATR1 POLR2D MRPL44 LSS RPF2 INTS3 RAB39B LRRC47 CUL1 DHX9 EIF4B TNFAIP1 ROCK1 VEZF1 BTBD3 TBL1X CHCHD3 NIP7 MARCKS NT5C3A PNRC2 NCS1 COPS5 TVP23B OPTN EAPP MYO3A UGDH DENR SNX27 DNMT1 NFIX PIGA SRSF10 HCN2 DCAF15 ZFC3H1 ARRDC3 MYCBP2 MRPS5 JUNB HOOK2 TMEM245 IDI1 ACTR1B FNDC3A SPIRE1 LAMTOR1 DIAPH1 PPIL2 ASB6 CEBPG GTF3C4 SLC20A2 IQSEC1 PSMA7 TUBG1 NCBP1 IL6ST MRPS27 GLYR1 RPL18 PPM1L SMOC1 VAMP3 CDK5R1 PARL PAPSS1 BEX1 SCAMP1 GOLIM4 ANAPC5 GLRX5 ST14 HIF1AN NCKIPSD SLC46A1 RRM1 COX5B MFSD12 NIPA1 UBA2 RAB12 SERPINE1 C18orf25 B4GALT3 EWSR1 COPB1 CBY1 HTRA2 DLD PPFIA1 HBS1L DNAJB12 HLA-B ARFGEF1 HNRNPA3 QSER1 HNRNPLL RHOBTB3 EIF4A2 POFUT1 LYSMD1 CUX1 PTP4A1 PPP1R14C ARID4B UBE2V2 FKBP9 DDR1 RNF157 LMNA ZNF12 SLC25A38 CEBPB SUPT7L RANBP2 CAST DDX3X DHX36 DAAM1 B3GAT3 MBOAT2 TRAP1 ATMIN APP RPS6KA3 SMAD5 CDK10 USP3
Essential genes	109	LCORL PCSK1 POU3F1 FEV HES1 ISL1 TM4SF4 AIRE SYT13 FOXA2 HNF6 RFX3 BCHE MEIS2 GCK IRS1 GHRL SMAD9 MAFA PROX1 SOX9 GC SIX2 SHH ENTPD2 CHN2 IGFBP2 SST PAX2 LMX1A PCSK2 SMARCA1 PPY INS ARX HHEX ADCYAP1 PFKFB2 IAPP NKX2-3 THSD7A EGFR AQP3 PDX1 PDLIM4 GLIS3 CPA3 NKX6-2 ID2 GSH2 GLUT1 PGR RFX6 KRT19 GSH1 HNF1B TGFBR3 GABRG2 UCN3 NOVA1 DLK1 NKX2-2 ETV1 REG3A BMP5 ZNF503 RFX2 HEX PTGER3 ERBB4 RFX5 PAX4 NEUROG3 TFCP2L1 SIX3 PTGFR LEPR CRYBA2 NPTX2 RFX1 AMY2A KLH41 SOX17 NKX6-3 NEUROD1 SLITRK6 EGR3 PTF1A HES3 CPA1 HNF1A PRG4 TTR RBP4 IGF2 ID4 MIST1 LDB2 IRX2 CARTPT HNF4A SERTM1 CDKN1C LMO3 GCG FRZB LMX1B GATA4 MNX1

Essential genes only

Names	total	elements
miR-148a-3p miR-375 miR-7-5p	2	IRS1 SOX4
miR-375 miR-7-5p	1	GATA6
miR-148a-3p miR-375	1	PSIP1
miR-148a-3p miR-7-5p	1	ERRFI1
miR-375	9	LOXL4 PLCE1 ERBB4 HMGB3 RFX7 EGR3 RFX3 NKX6-1 EHF
miR-7-5p	10	RGS4 EGFR IRS2 CPA4 IDE IGFBP4 KRT18 PIR KRT7 PAX6
miR-148a-3p	9	PTGER3 BHLHE41 LEPR MEIS1 RGS2 PGR LCORL MAFB HHEX

Names	total	elements
Essential genes miR-148a-3p miR-375 miR-7-5p	2	IRS1 SOX4
Essential genes miR-375 miR-7-5p	1	GATA6
Essential genes miR-148a-3p miR-375	1	PSIP1
Essential genes miR-148a-3p miR-7-5p	1	ERRFI1
Essential genes miR-375	9	PLCE1 ERBB4 RFX3 NKX6-1 EHF LOXL4 HMGB3 RFX7 EGR3
Essential genes miR-7-5p	10	IRS2 CPA4 IDE IGFBP4 RGS4 PIR KRT18 PAX6 EGFR KRT7
Essential genes miR-148a-3p	9	PTGER3 LEPR RGS2 LCORL MEIS1 HHEX BHLHE41 PGR MAFB
Essential genes	99	SYT13 NEUROG3 HEX FOXA2 HNF6 NEUROD1 PDLIM4 KRT19 TFCP2L1 PROX1 CPA1 DLK1 LMO3 CRYBA2 BCHE MEIS2 SOX9 NPTX2 PPY GC GSH1 HNF1A NKX2-2 GLIS3 INS GCK RFX5 MIST1 LDB2 HNF1B CPA3 TGFBR3 PAX2 GHRL ETV1 PCSK1 REG3A LMX1A SMAD9 SIX2 PAX4 RFX1 ARX IRX2 PRG4 TTR GABRG2 GCG PCSK2 AMY2A BMP5 KLH41 SHH RBP4 SLITRK6 ADCYAP1 PFKFB2 POU3F1 NKX6-2 PTF1A CARTPT FRZB HNF4A IGF2 UCN3 FEV IAPP MAFA HES1 NOVA1 ID4 NKX2-3

		ZNF503 ISL1 HES3 ID2 GSH2 LMX1B GATA4 MNX1 THSD7A GLUT1 ENTPD2 SIX3 SOX17 PTGFR SMARCA1 CHN2 SERTM1 RFX2 IGFBP2 TM4SF4 RFX6 NKX6-3 AQP3 AIRE CDKN1C PDX1 SST
